# Upregulation of RGS2: a new mechanism for pirfenidone amelioration of pulmonary fibrosis

**DOI:** 10.1186/s12931-016-0418-4

**Published:** 2016-08-22

**Authors:** Yan Xie, Haihong Jiang, Qian Zhang, Suneet Mehrotra, Peter W. Abel, Myron L. Toews, Dennis W. Wolff, Stephen Rennard, Reynold A. Panettieri, Thomas B. Casale, Yaping Tu

**Affiliations:** 1 Department of Pharmacology, Creighton University School of Medicine, 2500 California Plaza, Omaha, NE 68178 USA; 2 Department of Pharmacology and Experimental Neuroscience, University of Nebraska Medical Center, Omaha, NE USA; 3 Department of Biomedical Sciences, University of South Carolina School of Medicine Greenville, Greenville, SC USA; 4 Division of Pulmonary, Critical Care, Allergy and Sleep Medicine, University of Nebraska Medical Center, Omaha, NE USA; 5 Clinical Discovery Unit, AstraZeneca, Cambridge, UK; 6 Pulmonary, Allergy and Critical Care Division, Airways Biology Initiative, University of Pennsylvania, Philadelphia, PA USA; 7 Department of Internal Medicine, University of South Florida School of Medicine, Tampa, FL 33620 USA

**Keywords:** RGS2, Pirfenidone, Bleomycin, Early response genes, Human fetal lung fibroblasts, Primary human pulmonary fibroblasts, Proliferation, Fibroblast to myofibroblast differentiation, Knockout, Idiopathic pulmonary fibrosis

## Abstract

**Background:**

Pirfenidone was recently approved for treatment of idiopathic pulmonary fibrosis. However, the therapeutic dose of pirfenidone is very high, causing side effects that limit its doses and therapeutic effectiveness. Understanding the molecular mechanisms of action of pirfenidone could improve its safety and efficacy. Because activated fibroblasts are critical effector cells associated with the progression of fibrosis, this study investigated the genes that change expression rapidly in response to pirfenidone treatment of pulmonary fibroblasts and explored their contributions to the anti-fibrotic effects of pirfenidone.

**Methods:**

We used the GeneChip microarray to screen for genes that were rapidly up-regulated upon exposure of human lung fibroblast cells to pirfenidone, with confirmation for specific genes by real-time PCR and western blots. Biochemical and functional analyses were used to establish their anti-fibrotic effects in cellular and animal models of pulmonary fibrosis.

**Results:**

We identified Regulator of G-protein Signaling 2 (RGS2) as an early pirfenidone-induced gene. Treatment with pirfenidone significantly increased RGS2 mRNA and protein expression in both a human fetal lung fibroblast cell line and primary pulmonary fibroblasts isolated from patients without or with idiopathic pulmonary fibrosis. Pirfenidone treatment or direct overexpression of recombinant RGS2 in human lung fibroblasts inhibited the profibrotic effects of thrombin, whereas loss of RGS2 exacerbated bleomycin-induced pulmonary fibrosis and mortality in mice. Pirfenidone treatment reduced bleomycin-induced pulmonary fibrosis in wild-type but not RGS2 knockout mice.

**Conclusions:**

Endogenous RGS2 exhibits anti-fibrotic functions. Upregulated RGS2 contributes significantly to the anti-fibrotic effects of pirfenidone.

**Electronic supplementary material:**

The online version of this article (doi:10.1186/s12931-016-0418-4) contains supplementary material, which is available to authorized users.

## Background

Idiopathic pulmonary fibrosis (IPF) is a chronic and ultimately fatal disease characterized by a progressive decline in lung function [1, 2]. In the US, IPF affects over 150,000 people, and 40,000 patients die from IPF each year [3, 4]. It is widely thought that IPF causes loss of alveolar epithelium and accumulation of fibroblasts and myofibroblasts that in turn result in collagen deposition and fibrosis. These changes cause disruption of the gas permeability barrier and stiffen lung tissue, thereby impairing ventilation of nearby normal lung tissue [1, 5, 6]. IPF patients eventually experience dyspnea at rest and, without a lung transplant, have a 5-year average survival rate of only 20 % [3, 4].

A wide range of therapies have failed in IPF clinical trials [7, 8]. Pirfenidone (PFD) is an orally administered small molecule drug with anti-fibrotic and anti-inflammatory activities [9–12]. It slows the progressive loss of lung function in IPF and was recently approved by the U.S. Food and Drug Administration (FDA) to treat IPF [13–16]. Unfortunately, the therapeutically effective dose of PFD is very high (>30 mg/kg, daily) [17, 18], which can cause significant side effects such as gastro-esophageal reflux, phototoxicity, dizziness, and fatigue, thereby limiting its therapeutic effectiveness [16]. Understanding the molecular mechanisms that contribute to PFD effects could improve its safety and efficacy. Activated fibroblasts/myofibroblasts are key pathological features of IPF and are critical effector cells associated with the progression of fibrosis [5, 6, 19, 20]. Several studies have indicated that PFD reduces growth factor-driven fibroblast proliferation, differentiation, and extracellular matrix production [10, 21]. However, the specific molecular mechanisms whereby PFD produce these diverse effects to achieve its clinical benefits in IPF remain unknown [15].

In the studies presented here, we used the GeneChip microarray method to identify RGS2 as an early response gene elevated in response to PFD treatment of human lung fibroblast cells. RGS2 selectively inhibits the magnitude and duration of G_q_ protein-coupled receptor-induced signaling [22–24]. G protein-coupled receptors (GPCRs) have been shown to play important roles in the pathogenesis of chronic lung diseases including IPF [25–28]. There is also evidence that RGS2 is functionally important in regulating the pathogenesis of fibrosis. For example, RGS2 was found to inhibit the progression of kidney fibrosis following unilateral ureteral obstruction in mice [29]. We recently found that mice lacking RGS2 exhibit increased peribronchial fibrosis in an acute mouse model of asthma induced by administration of intranasal interleukin 13 (5 μg/day) for 3 days [30]. However, the role of RGS2 in the onset and progression of IPF remains unknown. Using cellular and animal models, we demonstrate here that endogenous RGS2 exhibits anti-fibrotic functions and that early upregulation of RGS2 contributes to PFD amelioration of pulmonary fibrosis.

## Methods

### Reagents and cells

PFD (5-methyl-1-phenyl-2-(1H)-pyridone) and human thrombin were purchased from Sigma-Aldrich (St. Louis, MO). Unless indicated otherwise, all other reagents were from Sigma-Aldrich or Thermo Fisher Scientific (Waltham, MA). The human fetal lung fibroblast cell line (HFL1) was purchased from ATCC (CCL-153™, Manassas, VA). Primary human lung fibroblasts were isolated and cultured from lung tissues obtained during open lung biopsy from patients who were undergoing lung transplantation or at time of death. Control primary human lung fibroblast (CPHLF) cell lines were established by Dr. Reynold Panettieri’s lab (University of Pennsylvania) from three patients with brain-related disease but no history of pulmonary fibrosis. Diseased primary human lung fibroblast (DPHLF) cell lines were established by Dr. Carol Feghali-Bostwick (Medical University of South Carolina) [31, 32] from two patients with IPF who had a confirmed diagnosis based on the criteria established by the American Thoracic Society. The biopsies had no identifiers, and the protocols for cell isolation were approved by the respective universities’ Institutional Review Boards.

### Cell culture and drug treatments

Cells were routinely cultured at 37 °C with 5 % CO_2_ in 1:1 mixture of Dulbecco's Modified Eagle's Medium (DMEM) and Ham's F-12 Nutrient Mixture (F12) supplemented with 10 % FBS and were used at passage <10 for experiments. For dose–response experiments, human lung fibroblast cells were seeded into 12-well plates, starved in serum-free DMEM/F12 medium for 24 h, and then treated with the indicated concentrations of PFD for the indicated times.

### Quantitative PCR

Total RNA extraction and quantitative real-time PCR were conducted as described previously [33]. The RNA samples were treated with DNase I (Thermo Scientific, Waltham, MA) to remove contaminating genomic DNA prior to experiments. The housekeeping gene β-actin plasmid was purchased from ATCC (MGC-10559). Human RGS2 was constructed into pcDNA3.1 (Invitrogen Life Technology) [30]. For RGS2, standard curves were generated by plotting the threshold cycle (C_T_) against the natural log of the copy number of plasmid molecules. The equations from the graphs were used to calculate the copy numbers of cDNA molecules present in the unknown samples based on the corresponding C_T_ values. For connective tissue growth factor (CTGF), standard curves were generated by plotting the C_T_ against serial dilutions of sample. The primers for CTGF were: 5’-CAGCATGAAGACATACCGAGC-3’ and 5’-GACAGTTGTAATGGCAGGCAC-3’.

### GeneChip microarray

Total RNA of control and PFD-treated HFL1 cells pooled from three individual experiments were analyzed by the Genome Sequencing Facility, University of Kansas Medical Center using Human Gene 2.0 ST Arrays (Affymetrix, Santa Clara, CA) and standard Affymetrix protocols [34]. GeneChip microarray analysis was replicated with two different pooled samples and the results were expressed as the ratio of PFD-treated vs. vehicle control.

### Western blot analysis

Cell protein extraction and western blot were conducted as described previously [30, 35]. Protein was extracted from cells using 1x RIPA lysis buffer (Santa Cruz, Dallas, TX). Samples were electrophoresed and subjected to western blot using primary antibodies against RGS2 (Proteintech, Chicago, IL) and β-actin. IRDye-800 secondary antibodies (LI-COR, Lincoln, NE) were used to capture images with a LI-COR Odyssey.

### Overexpression of recombinant RGS2 in HFL1 cells

The medium of HFL1 cells (50 % confluence) was replaced with growth medium containing human Adenovirus Type5 expressing RGS2 under the ubiquitin C promoter with co-expression of an mCherry reporter under a separate cytomegalovirus promoter (Ad-RGS2/mCherry) (SignaGen Laboratories, Rockville, MD) at a multiplicity of infection of 50 plaque-forming units. Cells infected with adenovirus expressing mCherry reporter (Ad-mCherry) were used as a control. The cells were incubated for 24 h at 37 °C and re-seeded for various experiments.

### Measurement of intracellular Ca^2+^ ([Ca^2+^]_i_)

HFL1 cells seeded into 96-well plates at 1 × 10^5^cells/well were cultured in serum-free medium for 24 h. Thrombin-induced changes in intracellular Ca^2+^ concentration were measured with the Fluo-8 No Wash Calcium Assay kit (Abcam, Cambridge, MA) according to kit instructions. The plates were transferred to a FLEX Station II benchtop scanning fluorometer chamber (Molecular Devices, Sunnyvale, CA). The cells were excited at 490 nm and Ca^2+^-bound fluo-8 emission was recorded at 525 nm. The fluo-8 fluorescence was expressed as F_max_/F_0_ where F_max_ was the maximum and F_0_ was the baseline fluorescence measured.

### Cell proliferation assay

HFL1 cells were treated with thrombin (1 U/ml, Sigma-Aldrich, St. Louis, MO) for 6 h and then labeled with 10 μM 5-bromo-2’-deoxyuridine (BrdU) (BD Pharmigen, San Jose, CA) for 18 h. Cells were stained for nuclei and BrdU using 4’,6-diamidino-2-phenylindole (DAPI) and anti-BrdU antibody (Cell Signaling, Danvers, MA) [36]. Results are expressed as the percentage of DAPI-stained cells that were also BrdU-positive.

### Immunofluorescence staining for α-smooth muscle actin

HFL1 cell α-smooth muscle actin (α-SMA) was visualized with an anti-α-actin primary antibody and an Alexa Fluor 488-labeled secondary antibody [37]. The results are expressed as the percentage of DAPI-stained cells that also have clear α-SMA–positive stress fibers.

### Gel contraction assay

HFL1 cells (2.5×10^5^ cells/ml) were suspended in type I collagen from rat tail tendon (1.25 mg/ml, BD Bioscience, Bedford, MA). The collagen-cell suspension was added to 24-well plates (300 μl /well) and allowed to polymerize for 45 min at 37 °C. After incubating with DMEM containing 10 % fetal bovine serum (FBS) for 4 h, the medium from the polymerized gels was changed to serum-free medium. Polymerized gels were incubated overnight and were then stimulated with thrombin (1 U/ml) for 24 h. To initiate collagen gel contraction, polymerized gels were gently released from the underlying culture plate and digitally photographed. The gel areas were analyzed using Image-Pro Plus 6.0 (Media Cybernetics, Rockville, MD) and expressed as arbitrary area units [37].

### Measurement of collagen production and deposition

The amount of lung collagen was measured using a Sirius red total collagen detection kit (Chondrex, Redmond, WA) as described [38]. Briefly, to determine collagen production from fibroblasts in vitro, HFL1 cells seeded into 6-well plates (5 × 10^5^cells/well) were starved for 24 h and then stimulated with 1 U/ml thrombin for another 24 h. Cells were harvested and the amount of collagen in cell lysates was determined. To measure collagen deposition in vivo, mouse lung tissues were minced and subjected to the same analysis of the amount of collagen [30].

### Bleomycin-induced mouse pulmonary fibrosis and inhalation delivery of PFD

All animal studies were approved by the Institutional Animal Care and Use Committee at Creighton University. Age- (~11 weeks) and weight- (~20 g) matched RGS2 wild-type (+/+) and knockout (−/−) C57BL/6 J mice [39] were obtained by in-house breeding and are descendants from the original breeding pairs provided by the University of Texas Southwestern Medical Center with approval from the originator, Dr. David Siderovski (West Virginia University).

Anesthetized mice were administered a single dose of bleomycin sulfate dissolved in 0.9 % saline by intratracheal instillation with an IA-1C MicroSprayer aerosolizer (PennCentury, Wyndmoor, PA) to induce pulmonary fibrosis [18, 40]. Pulmonary fibrosis was evaluated 21 days later. A lower dose of bleomycin (1.0 U/kg body weight) was used for comparing alterations in lung function and histology between RGS2+/+ and RGS2−/− mice. To compare long-term survival rates, 2.0 U/kg of bleomycin was administered.

To examine therapeutic effects of PFD, mice were administrated bleomycin (1.5 U/kg body weight) as described above and divided into four groups (4 mice/group): bleomycin in RGS2+/+, bleomycin + PFD in RGS2+/+, bleomycin in RGS2−/−, bleomycin + PFD in RGS2−/−. One week after bleomycin administration, PFD solution was administered using a nose-only aerosol exposure tower (Data Sciences International, St. Paul, MN). Aerosol was delivered with the Aeroneb Lab Micropump Nebulizer. Mice received daily 20-minute exposures to 0.4 ml of aerosolized 5 mM PFD or saline (vehicle control) for 14 successive days. Actual administered doses were calculated according to the following formulas [41]:

$$ Estimation\  of\  inhaled\  volume\  per\  mouse\ (V1) = \left(N/VA\right) \times TV \times RR \times T $$

$$ Estimation\  of\ PFD\  administered\  dose\ D = \left(C \times V1\right)/W $$

In these formulas, N is nebulization rate (300 ml/min), VA is air flow (0.2 L/min), TV is tidal volume (estimated at 170 ml), RR is respiratory rate (estimated at 150 breaths/min), T is the duration of treatment, C is PFD concentration (926 mg/L), and W is mouse weight. Based on these parameters, the estimated administered dose was 0.35 mg PFD/kg daily.

### Mouse lung function analysis

Mouse lung dynamic compliance (C_dyn_) was recorded using a FinePointe system (Buxco, St. Paul, MN) as previously described [30, 35].

### Histologic analysis

Sections of paraformaldehyde-fixed mouse lungs were analyzed by hematoxylin and eosin (H&E) or Masson’s trichrome staining to assess fibrotic changes in the lungs [30, 42]. Photos of 20 fields from multiple sections of each mouse were taken at 100X magnification and scored separately by the modified Ashcroft method (score range 0–8) [43, 44]. The pulmonary fibrosis histopathology score of each mouse is expressed as the mean score of 20 photos.

### Statistical analysis

Data are expressed as means ± SEM. Groups were compared using Student’s *t* test for unpaired observations or two-way ANOVA with the Bonferroni correction for multiple comparisons. *p* < 0.05 was taken as statistically significant.

## Results

### RGS2 is an early response gene elevated by PFD treatment of HFL1 cells

**Fig. 1:**
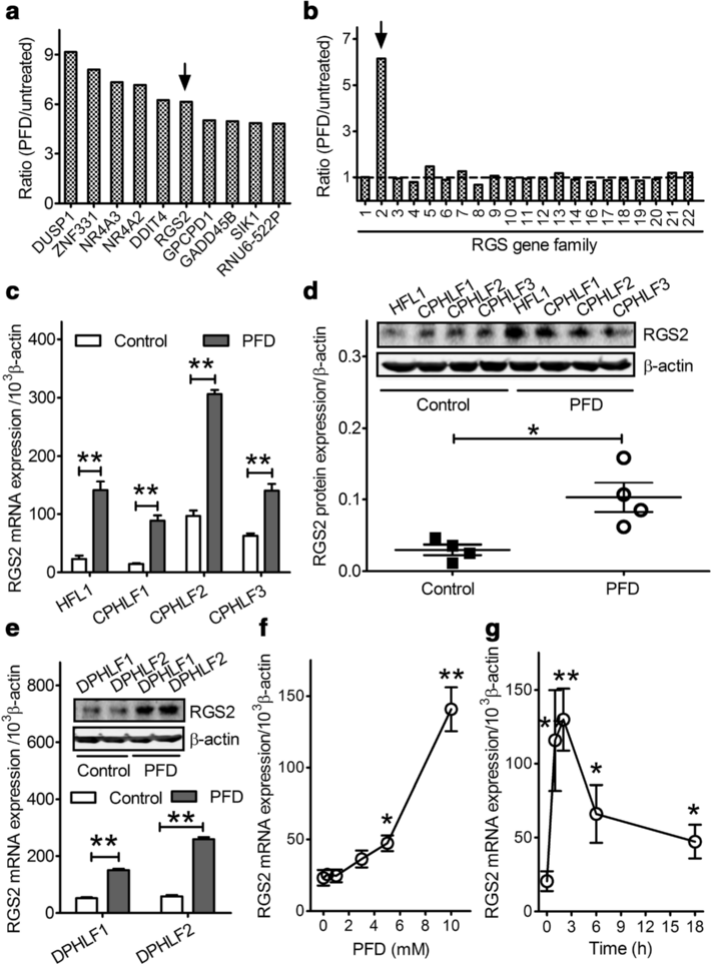
Identification of RGS2 as an early response gene to PFD treatment in human lung fibroblasts. Genechip microarray revealed PFD–induced upregulated genes, with the top ten shown (**a**) and selective upregulation of RGS2 in the RGS gene family in human fetal lung fibroblast (HFL1) cells (**b**). Data shown are the means of two Genechip microarray assays using different pooled total RNA. The results are expressed as the fold change calculated as the ratio of the two groups. HFL1, three control primary human lung fibroblast (CPHLF1-3) (**c** and **d**), and two diseased primary human lung fibroblast (DPHLF1-2) (**e**) cell lines were treated with saline (control) or 10 mM PFD for 2 h and then processed for real-time PCR (**c**, **e**) and western blot analyses (**d**, **e**). The results of real-time PCR were normalized as RGS2/β-actin × 1000 and plotted as means ± SEM (*n* = 4). A blot representative of three experiments is shown (**d** and **e**, insets). Mean RGS2 band density was normalized to β-actin ± SEM. HFL1 cells were treated with various concentrations of PFD (0–10 mM) for 2 h (**f**) or with 10 mM PFD over a time course of 18 h (**g**). Cells were harvested for real-time PCR analysis of RGS2 and β-actin mRNA levels. The results are shown as means ± SEM (*n* = 4). * *p* < 0.05, ** *p* < 0.01 vs. control

The transcriptome profiles of control versus PFD-treated HFL1 cells were determined by GeneChip microarray analysis. This analysis of 48,226 human transcripts revealed 76 genes that were upregulated by at least 2-fold within the first 2 h of treatment with 10 mM PFD (Additional file 1: Table S1). The 10 most highly upregulated genes are presented in Fig. 1a. RGS2 was among the top six genes, with a 6-fold upregulation after PFD treatment. Interestingly, RGS2 was the only one among 21 members of the RGS gene family to be significantly upregulated in HFL1 cells (Fig. 1b). Because of our prior work and strong interest in RGS proteins, our further studies focused only on RGS2.

### RGS2 mRNA and protein expression levels are increased by PFD in HFL1 and primary human lung fibroblast cells

Quantitative RT-PCR analysis confirmed that 10 mM PFD treatment increased RGS2 mRNA levels in HFL1 cells and three control primary human lung fibroblast (CPHLF) cell lines (Fig. 1c). As expected, western blots confirmed that PFD treatment for 2 h also increased RGS2 protein levels in these fibroblast cells by 3.5-fold (Fig. 1d). We also investigated whether PFD induced RGS2 expression in diseased primary human lung fibroblast (DPHLF) cell lines established from patients with IPF. As shown in Fig. 1e, treatment with 10 mM PFD increased RGS2 mRNA and protein levels by 4- and 3-fold, respectively (Fig. 1e and inset). Quantitative RT-PCR analysis of HFL1 cells showed that RGS2 mRNA induction by PFD occurred in a concentration-dependent manner (Fig. 1f) and achieved statistical significance at concentrations of 5 and 10 mM (*p* < 0.05); 10 mM PFD caused a 6-fold induction of RGS2 mRNA. RGS2 mRNA induction occurred at all time points tested from 1 to 18 h after 10 mM PFD treatment; peak levels of 6-fold were seen 2 h after treatment and gradually declined, with 2.5-fold upregulation at 18 h (Fig. 1g).

### RGS2 protein attenuates thrombin-induced increase of [Ca^2+^]_i_ in HFL1 cells

**Fig. 2:**
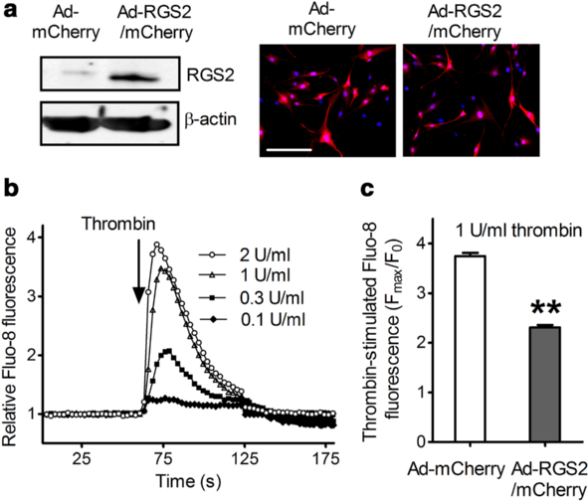
Increase of RGS2 attenuates thrombin-induced increase of [Ca^2+^]_i_ in HFL1 cells. HFL1 cells were infected with Ad-mCherry or Ad-RGS2/mCherry for 24 h. **a** RGS2 and mCherry expression was assessed by western blots (*left panel*) and fluorescence microscopy (*right panel*), respectively. Infection efficiency was approximately 90 % based on the percentage of cells expressing mCherry (scale bar = 50 μm). **b** Thrombin induced a dose-dependent increase of [Ca^2+^]_i_ in un-infected HFL1 cells. **c** Thrombin-induced [Ca^2+^]_i_ increase in HFL1 cells infected with Ad-mCherry or Ad-RGS2/mCherry. Cells pre-loaded with Fluo-8 were stimulated with various concentrations of thrombin (0.1–2 U/ml) (**b**) or 1 U/ml of thrombin (**c**). The increase of Fluo-8 fluorescence from four experiments is plotted as means ± SEM. ** *p* < 0.01 as compared to control untreated cells

The serine protease thrombin activates G_q_-coupled proteinase-activated receptor 1 (PAR1) to promote fibroblast proliferation and differentiation into a myofibroblast phenotype, contributing to development of pulmonary fibrosis [28, 37]. Previous studies have shown that thrombin stimulation of proliferation is dependent on PAR1-mediated increase of [Ca^2+^]_i_ in many cell types [45, 46]. Because RGS2 functions as a selective modulator of G_q_-mediated signaling [22–24], the effects of RGS2 expression on thrombin-induced increases of [Ca^2+^]_i_ in HFL1 cells were examined. RGS2 protein was increased by about 6-fold in HFL1 cells with adenovirus-expressing RGS2 and mCherry reporter in comparison to control cells with the mCherry-expressing adenovirus alone (Fig. 2a). Thrombin induced a dose-dependent increase of [Ca^2+^]_i_ in the mCherry-alone control HFL1 cells (Fig. 2b). As compared with these control cells, overexpression of RGS2 significantly attenuated the thrombin (1 U/ml)-induced increase in [Ca^2+^]_i_ in HFL1 cells from 3.75 ± 0.07 to 2.31 ± 0.05 (Fig. 2c, *p* < 0.01).

### RGS2 protein inhibits thrombin-induced proliferation of HFL1 cells

**Fig. 3:**
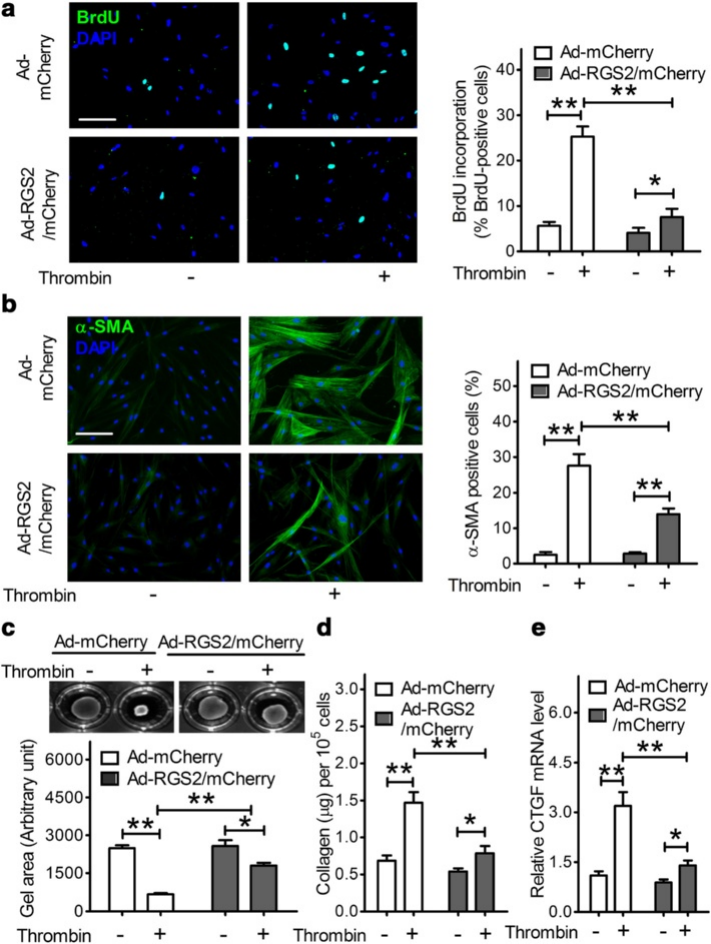
RGS2 suppresses the profibrotic effects of thrombin in HFL1 cells. HFL1 cells were infected with Ad-mCherry or Ad-RGS2/mCherry for 24 h. Cells were then treated without or with thrombin (1 U/ml) for 24 h. **a** HFL1 cell proliferation was assessed by BrdU incorporation assays. *Left panel*: representative staining images of BrdU (*green*) incorporation and DAPI (*blue*) indicated the location of nuclei under immunofluorescence microscopy (scale bar = 50 μm). *Right panel*: quantitative analysis for each treatment was performed by counting the percentage of DAPI-stained cells that were also BrdU-positive. Data are means ± SEM from at least 100 fibroblast cells. **b** HFL1 cell differentiation was assessed by immunofluorescence staining for α-SMA. *Left panel*: representative staining images of α-SMA (*green*) and DAPI (*blue*) indicated the location of nuclei under immunofluorescence microscopy (scale bar = 50 μm). *Right panel*: The results are expressed as the percentage of total DAPI-stained cells that also had clear α-SMA–positive stress fibers. Data are means ± SEM from at least 100 fibroblast cells. **c** HFL1 cell contraction was assessed by collagen gel contraction assays. Top: Images of collagen gels containing 2.5×10^5^ cells/ml without and with thrombin stimulation for 24 h. Bottom: Results are expressed as the mean gel area ± SEM from at least three separate experiments. **d** The amount of collagen in HLF1 cell lysates was measured by the Sirius *red* collagen assay. Results are expressed as μg of collagen per 10^5^ cells. Data are means ± SEM (*n* = 3). **e** HFL1 cells infected with Ad-mCherry or Ad-RGS2/mCherry were treated without or with 1 U/ml of thrombin for 3 h and then processed for real-time PCR. The results of real-time PCR were normalized as CTGF/β-actin and plotted as means ± SEM (*n* = 3). * *p* < 0.05, ** *p* < 0.01 between groups

We next investigated whether increases in RGS2 expression functionally attenuate thrombin-stimulated cell proliferation. BrdU incorporation assays showed that the proliferation rate (% BrdU-positive cells) in control HFL1 cells was increased from a basal level of 5.6 ± 0.9 % to 25.3 ± 2.2 % (*p* < 0.01) following thrombin (1 U/ml) treatment for 24 h (Fig. 3a). In contrast, the basal proliferation rate of HFL1 cells overexpressing RGS2 and mCherry was 4.1 ± 1.1 % and this value was increased to 7.6 ± 1.8 % by thrombin (Fig. 3a). Thus, overexpression of RGS2 attenuated the thrombin-stimulated cell proliferation by about 70 % (*p* < 0.01).

### RGS2 protein inhibits thrombin-induced differentiation of HFL1 cells

Fibroblasts treated with thrombin acquire a more contractile phenotype characterized by elevated α-SMA [34]. As shown in Fig. 3b (left panel), HFL1 cells treated with thrombin displayed abundant stress fibers that stained intensely for α-SMA. The percentage of α-SMA staining-positive HFL1 cells was significantly increased in both mCherry alone and mCherry plus RGS2 cells after stimulation for 24 h with thrombin. However, overexpression of RGS2 attenuated the thrombin-stimulated increase in α-SMA expression-positive cells by about half (14.0 ± 1.6 % vs. 27.6 ± 3.2 %; Fig. 3b, right, *p* < 0.05). Collagen gel contraction assays demonstrated that RGS2 attenuation of α-SMA expression has functional consequences. Thrombin stimulation of HFL1 cells expressing mCherry caused a 73 % reduction in gel size as compared to a less than 30 % reduction in gel size when HFL1 cells expressing RGS2 and mCherry were stimulated with thrombin (Fig. 3c). As expected, treatment with 1 U/ml of thrombin for 24 h increased collagen production in HFL1 cells, by 2.2-fold (0.68 ± 0.07 vs. 1.47 ± 0.14 μg per 10^5^ cells, *p* < 0.01). Overexpression of RGS2 reduced basal collagen production by about 20 % but significantly attenuated thrombin-stimulated collagen production in HFL1 cells by about half (0.78 ± 0.10 vs. 1.47 ± 0.14, *p* < 0.01) (Fig. 3d).

### RGS2 protein attenuates thrombin-stimulated CTGF mRNA expression in HFL1 cells

CTGF is an important mediator of fibrosis [47, 48]. A previous study showed that stimulation with thrombin rapidly induced CTGF expression that in turn increased extracellular matrix protein production in lung fibroblasts [49]. As shown in Fig. 3e, treatment with thrombin (1 U/ml) for 3 h caused a significant increase in CTGF mRNA levels in HFL1 cells transfected with mCherry (3.2 ± 0.41 vs. 1.1 ± 0.12, *p* < 0.01). Overexpression of RGS2 slightly reduced basal CTGF mRNA expression but significantly attenuated the thrombin-stimulated increase of CTGF mRNA expression by more than 50 % (1.4 ± 0.15 vs. 3.2 ± 0.41, *p* < 0.01).

### Treatment with PFD inhibits the profibrotic effects of thrombin in HFL1 cells

**Fig. 4:**
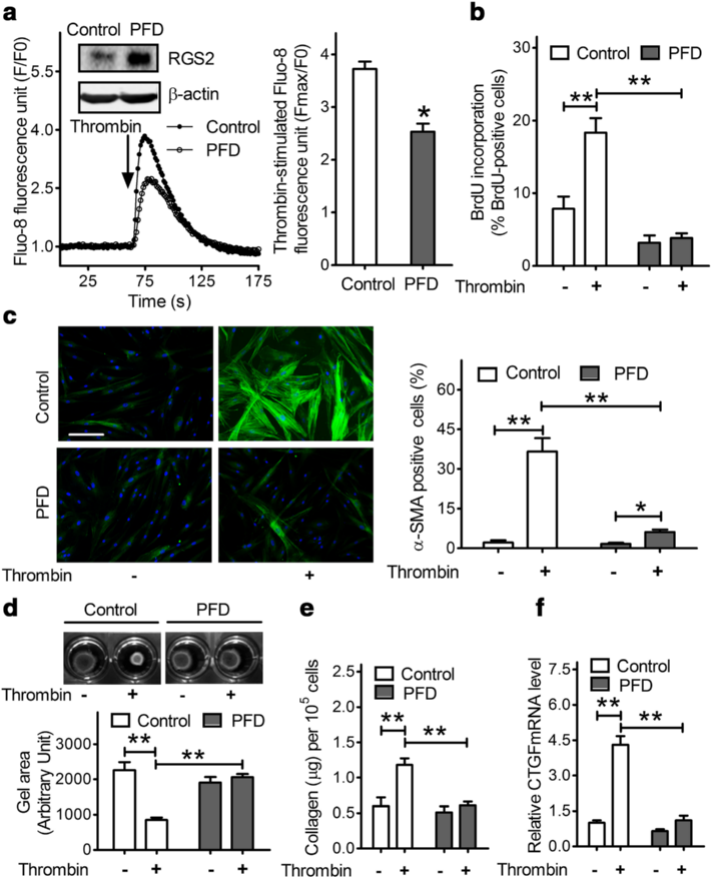
PFD treatment inhibits the profibrotic effects of thrombin in HFL1 cells. Cells were treated with vehicle control or 5 mM PFD for 24 h. **a** Thrombin (1 U/ml)-induced increase of [Ca^2+^]_i_ in control and PFD-treated HFL1 cells. The increase of Fluo-8 fluorescence from four experiments is plotted as means ± SEM. A representative western blot of RGS2 protein expression from three experiments is shown (**a**, inset). **b** HFL1 cell proliferation. Data are means ± SEM from at least 100 fibroblast cells. **c** HFL1 cell differentiation. *Left panel*: representative staining images of α-SMA (*green*) and DAPI (*blue*) (scale bar = 50 μm). *Right panel*: The results are expressed as the percentage of total DAPI-stained cells that also had clear α-SMA–positive stress fibers. Data are means ± SEM from at least 100 fibroblast cells. **d** HFL1 cell contraction. Top: Images of collagen gels containing 2.5×10^5^ cells/ml without and with thrombin stimulation for 24 h. Bottom: Results are expressed as the mean gel area ± SEM from at least three separate experiments. **e** The amount of collagen in HLF1 cell lysates. Data are means ± SEM (*n* = 3). **f** Treatment of PFD abolished thrombin-induced CTGF mRNA expression in HFL1 cells. Data were normalized as CTGF/β-actin and plotted as means ± SEM (*n* = 3). * *p* < 0.05, ** *p* < 0.01

Since PFD treatment induces RGS2 expression in human lung fibroblast, we next investigated whether PFD treatment also inhibits the profibrotic effects of thrombin. As shown in Fig. 4a (inset), treatment with 5 mM PFD increased RGS2 protein expression by 4-fold in HFL1 cells. As expected, the [Ca^2+^]_i_ transients induced by thrombin in HFL1 cells pretreated with PFD was significantly lower than that in cells pretreated with vehicle control (2.53 ± 0.15 vs. 3.72 ± 0.14, *p* < 0.05) (Fig. 4a). Thrombin significantly increased cell proliferation from 7.8 ± 1.7 % to 18.3 ± 2.0 % (*p* < 0.01) in control HFL1 cells. PFD treatment decreased the basal cell proliferation and completely abolished thrombin stimulation of HFL1 cell proliferation (Fig. 4b). It should be noted that PFD inhibition of cell proliferation was not due to cytotoxicity because trypan blue staining of cells showed no significant changes in cell viability with PFD treatment (data not shown).

We also investigated the effect of PFD treatment on thrombin-induced differentiation of HFL1 cells. As shown in Fig. 4c, thrombin treatment increased the percentage of α-SMA staining-positive cells in vehicle-treated control HFL1 cells by about 17-fold, from 2.2 ± 0.8 % to 36.6 ± 5.0 %, as compared to 3.5-fold increase (from 1.7 ± 0.4 % to 6.2 ± 0.9 %) in cells pre-treated with 5 mM PFD. Thus, the stimulatory effect of thrombin was largely attenuated by PFD treatment. In collagen gel contraction assays, thrombin stimulation of HLF1 cells caused a 54 % reduction in gel size (*p* < 0.01), whereas cells pretreated with PFD did not contract at all (Fig. 4d). Consistent with the results for differentiation, increased levels of collagen production (Fig. 4e) and CTGF mRNA expression (Fig. 4f) were detected when HFL1 cells were stimulated with thrombin, and these increases were largely abolished by PFD treatment. Thus, PDF treatment inhibits the profibrotic effects of thrombin in HFL1 cells.

### Loss of RGS2 augments the development of bleomycin-induced pulmonary fibrosis in mice

**Fig. 5:**
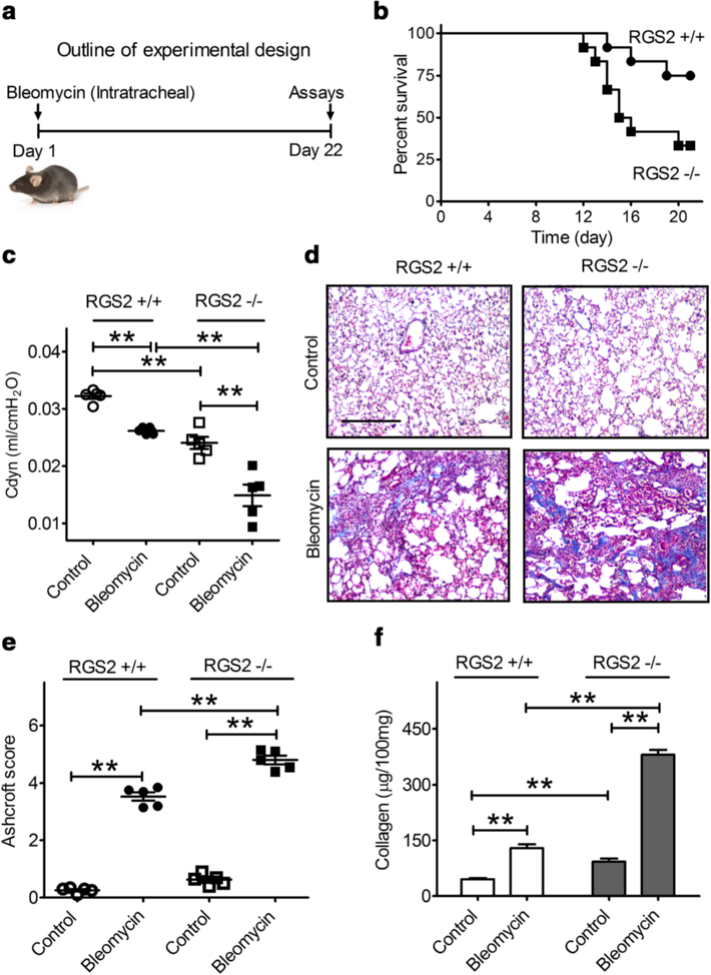
Loss of RGS2 arguments the development of bleomycin-induced pulmonary fibrosis in mice. **a** Outline of experimental design. On Day 1, bleomycin was administrated intratracheally into RGS2+/+ and RGS2−/− mice. On Day 22, dynamic lung compliance of mice was measured. **b** Loss of RGS2 augments the death rate in bleomycin (2 U/kg body weight)-induced mouse pulmonary fibrosis model. Kaplan-Meier survival curves were created for bleomycin-exposed RGS2+/+ and RGS2−/− mice (*n* = 12 per group). **c** Effects of bleomycin (1 U/kg) on dynamic lung compliance (C_dyn_) of RGS2+/+ and RGS2−/− mice. **d** The lungs were stained with trichrome to visualize collagen deposition (*blue*). Representative sections from each group are shown (scale bar = 200 μm). **e** Sections were scored by the modified Ashcroft method for overall fibrosis. Results are shown as means ± SEM (*n* = 5) and were analyzed using a two-sided *t*-test. **f** Total lung collagen content was measured by the Sirius red collagen assay. Results are expressed as μg of collagen per 100 mg of lung tissue. Data are means ± SEM (*n* = 5 mice). ** *p* < 0.01

The mouse bleomycin-induced lung fibrosis model has been widely used to explore both the pathogenesis of IPF and potential new therapies [18, 39]. We used this model to investigate the importance of RGS2 in regulation of the pathogenesis of IPF. RGS2 wild-type (+/+) and RGS2 knockout (−/−) mice (12 per group) were administered a high intratracheal dose of bleomycin (2 U/kg body weight) (Fig. 5a). By 21 days after exposure, 3 of the RGS2+/+ mice and 8 of the RGS2−/− mice had died. The Mantel-Cox log rank test showed that the survival rate of the RGS2−/− mice was significantly shorter than that of the RGS2+/+ mice (*p* = 0.03) (Fig. 5b).

Next, a lower bleomycin dose (1 U/kg) or saline control was instilled intratracheally into RGS2+/+ and RGS2−/− mice; this dose did not cause any mortality in either group. Dynamic lung compliance (inverse of lung stiffness) of these mice measured on Day 22 was significantly lower in control RGS2−/− mice compared with control RGS2+/+ mice (0.024 ± 0.001 vs. 0.032 ± 0.001, *p* < 0.01). Bleomycin induced a significant loss of lung compliance in both RGS2+/+ (0.026 ± 0.001 vs. 0.032 ± 0.001, *p* < 0.01) and RGS2−/− mice (0.015 ± 0.002 vs. 0.024 ± 0.001, *p* < 0.01). However, the loss in lung compliance caused by bleomycin treatment was 2 times greater in RGS2 −/− mice (38 %) than in RGS2+/+ mice (19 %) (Fig. 5c).

Histochemical staining with trichrome indicated that bleomycin administration induced deposition of collagen in both RGS2+/+ and RGS2−/− mice (Fig. 5d), but the collagen deposition was greater and its overall distribution was more widespread in RGS2−/− mice. Scoring the bleomycin-induced histological fibrosis by the Ashcroft method confirmed that pulmonary fibrosis was significantly increased in RGS2−/− mice as compared with RGS2+/+ mice (4.80 ± 0.15 vs. 3.53 ± 0.14, *n* = 5, *p* < 0.01) (Fig. 5e). Total lung collagen assays showed nearly two-fold increased basal levels of collagen in RGS2−/− mice when compared with RGS2+/+ mice (93.0 ± 8.2 vs. 45.3 ± 3.6 μg per 100 mg of lung tissue, *p* < 0.01), and bleomycin treatment further increased the RGS2−/− lung collagen to 380.6 ± 12.7 μg, more than three times higher than that in bleomycin-treated RGS2+/+ mice (129.2 ± 10.5 μg) (*p* < 0.01) (Fig. 5f).

### Local delivery of PFD ameliorates bleomycin-induced pulmonary fibrosis in RGS2+/+ but not in RGS2−/− mice

**Fig. 6:**
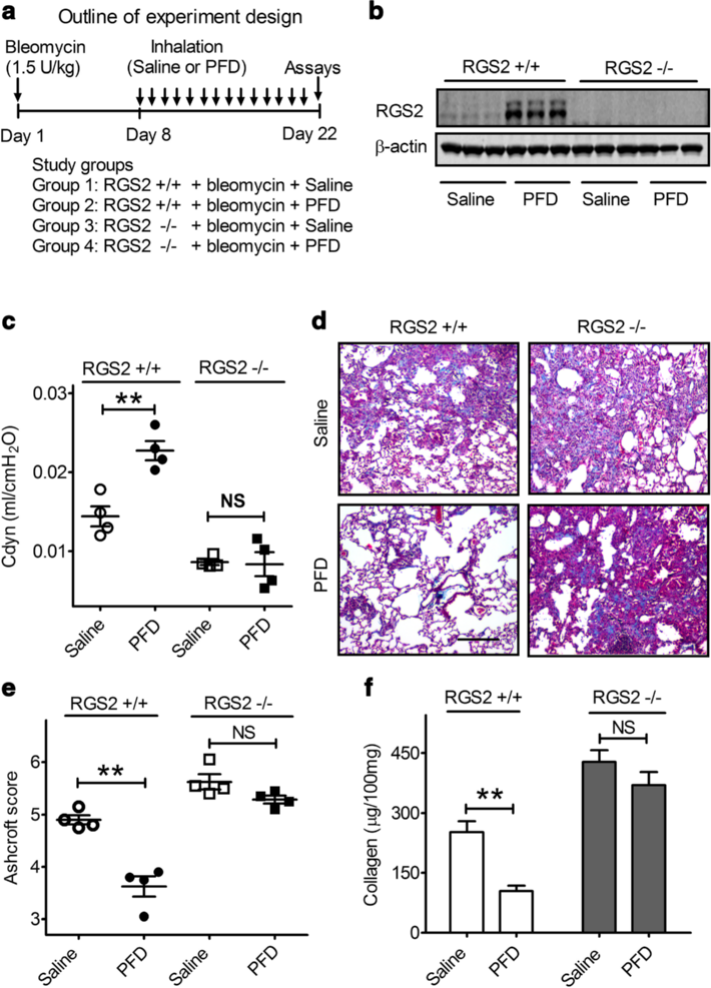
PFD ameliorates bleomycin-induced pulmonary fibrosis in RGS2+/+ but RGS2−/− mice. **a** Outline of experimental design. Bleomycin (1.5 U/kg body weight) was administrated intratracheally on Day 1. Mice were divided into four groups (4 mice/group): bleomycin in RGS2+/+, bleomycin + PFD in RGS2+/+, bleomycin in RGS2−/−, bleomycin + PFD in RGS2-/-. Treatment with PFD was started 1 week later and continued for 2 weeks. All mice received daily 20-min exposures to nebulized PFD solution or saline vehicle via a nose-only aerosolized delivery system. On Day 22, mice were tested in functional assays. **b** Representative western blot of RGS2 protein expression in lung homogenates of RGS2+/+ and RGS2−/− mice without or with PFD treatment. **c** C_dyn_ was measured using a Buxco FinePointe system. **d** The lungs were stained with trichrome to visualize collagen deposition (*blue*). Representative sections from each group are shown. **e** Sections were scored by the Ashcroft method for overall fibrosis (scale bar = 200 μm). **f** Total lung collagen content was measured by the Sirius *red* collagen assay. Results are expressed as μg of collagen per 100 mg of lung tissue. Data are means ± SEM (*n* = 4 mice). * *p* < 0.01, NS: not significant

We next tested the effectiveness of PFD to reduce bleomycin-induced fibrosis. One week after intratracheal administration of 1.5 U/kg bleomycin, mice were treated daily for 2 weeks with saline vehicle or PFD (0.35 mg/kg) via nose-only aerosolized delivery (Fig. 6a). Western blots showed that PFD treatment increased RGS2 expression in the lungs of RGS2+/+ mice, whereas no RGS2 protein was detected in RGS2−/− mice (Fig. 6b). In addition, RGS2+/+ mice treated with PFD exhibited significantly higher lung compliance than mice treated with vehicle (0.023 ± 0.001 vs. 0.014 ± 0.001, *p* < 0.01) (Fig. 6c), indicating that the bleomycin-induced loss of lung compliance was attenuated by PFD treatment. Administration of PFD also ameliorated the fibrotic lung lesions induced by bleomycin in RGS2+/+ mice, as shown by trichrome staining of collagen deposition (Fig. 6d). The Ashcroft score in bleomycin-treated RGS2+/+ mice was significantly reduced by PFD treatment, from 4.90 ± 0.09 to 3.62 ± 0.19 (Fig. 6e, *p* < 0.01). The collagen levels in the lungs of bleomycin-treated RGS2 +/+ mice were decreased by 60 % (n = 4, *p* < 0.01) (Fig. 6f). In contrast, in RGS2−/− mice, the bleomycin-induced changes in lung compliance and deposition of collagen were unaffected by PFD treatment (Fig. 6c–f). Thus, the elevated RGS2 expression induced by PFD treatment is crucial for PFD protection of mice against bleomycin-induced pulmonary fibrosis.

## Discussion

Although PFD is approved by the FDA for treatment of IPF, its cellular targets and molecular mechanisms of action remain poorly understood. In the present study, we took the novel approach of screening for genes whose expression changed rapidly upon exposure of HFL1 cells to PFD. This screen identified RGS2 as one of the genes most highly upregulated in response to short-term PFD treatment. Quantitative RT-PCR and western blot analyses confirmed PFD induction of RGS2 expression in five other primary human lung fibroblast cell lines isolated from patients without or with IPF, confirming that this is a general phenomenon. This induction of RGS2 expression is a previously unrecognized genomic response to PFD, and our further studies strongly suggest that RGS2 upregulation is a significant contributor to the pulmonary fibrosis protection induced by PFD.

RGS2 inhibits both the amplitude and duration of signals mediated by G_q_-coupled GPCRs [21–23]. Interestingly, several G_q_-coupled GPCRs and their ligands are important drivers of pulmonary fibrosis, including PAR1, lysophosphatidic acid receptor 1, and endothelin receptors [25–28]. Inhibition of these signals by RGS2 that is upregulated in response to PFD thus provides a mechanistic rationale for the beneficial effects of both PFD and RGS2 in terms of reducing fibrotic responses of lung fibroblasts. Indeed, when HFL1 cells were exposed to thrombin, a protease elevated in bronchial alveolar lavage fluid of IPF patients [50], PFD treatment or direct overexpression of recombinant RGS2 to levels similar to those induced by PFD treatment can trigger several anti-fibrotic responses in HFL1 cells. For example, PFD treatment or overexpression of RGS2 suppressed thrombin-induced proliferation and differentiation of HFL1 cells, key components of IPF [37]. Thrombin-induced collagen production, CTGF expression, and gel contraction were also inhibited by PFD treatment or RGS2 overexpression. Altogether, our study demonstrates for the first time that RGS2 is induced by PFD treatment and that it exhibits multiple anti-fibrotic effects. In addition, we found that PFD treatment or RGS2 overexpression significantly reduced thrombin-stimulated intracellular Ca^2+^ signaling. Since thrombin elevation of [Ca^2+^]_i_ promotes fibroblast proliferation and differentiation, our study provides a molecular mechanism to explain the anti-fibrotic effects of RGS2.

Interestingly, animal studies suggest an important role of RGS2 in regulation of the progression of IPF. Excess deposition of collagen by fibroblasts in the lung and decreased lung compliance are hallmarks of human IPF and of bleomycin-induced mouse pulmonary fibrosis [6, 11]. Our data showed a significant increase in collagen deposition in the lungs of bleomycin-treated RGS2 knockout (RGS2−/−) mice compared to that in bleomycin-treated wild-type (RGS2+/+) mice. Because the accumulation of collagen increases lung stiffness, lungs of bleomycin-treated RGS2−/− mice exhibited much lower compliance than lungs of similarly treated RGS2+/+ mice. The RGS2−/− mice also exhibited a decreased survival rate compared to RGS2+/+ mice, consistent with the high rate of mortality for IPF patients. Altogether, our study indicates that endogenous RGS2 itself plays a protective role and that loss of RGS2 augments the development of pulmonary fibrosis.

Animal studies provide further compelling support for the importance of PFD-induced RGS2 upregulation as part of PFD's anti-fibrotic action. PFD treatment significantly upregulated endogenous pulmonary RGS2 expression in RGS2+/+ mice. More importantly, PFD effectively reduced collagen deposition in the lung following bleomycin administration and ameliorated the bleomycin-induced decrease in lung compliance of RGS2+/+ mice. In marked contrast, PFD treatment had no effect in RGS2−/− mice, indicating that upregulated RGS2 is a crucial mediator of the anti-fibrotic effects associated with PFD treatment. Interestingly, RGS2 has been reported to be increased in IPF patient samples [51], which is consistent with the notion that upregulated RGS2 functions as a potential feed-back regulator of pulmonary fibrosis. Further investigation of the relative expression levels of RGS2 in patients with IPF following PFD treatment will be necessary to determine the clinical importance of RGS2 upregulation in PFD treatment of patients with IPF.

It should be noted that although western blot analyses showed upregulation of RGS2 protein expression in lungs of RGS2+/+ mice after PFD administration, the expression pattern of RGS2 induction in mouse lungs is unclear. We have performed immunohistochemical staining for RGS2 protein in paraffin-embedded mouse lung tissues. However, several RGS2 antibodies we tested showed positive staining in RGS2−/− mice. Thus, better antibodies will be needed to establish in which pulmonary cell types RGS2 up-regulation by PFD occurs in mice or in human patient samples. However, RGS2 is highly expressed in pulmonary fibroblast cells. In the bleomycin-induced pulmonary fibrosis mouse model, RGS2−/− mice had significantly increased pulmonary deposition of collagen compared to RGS2+/+ mice, and this collagen is predominately produced by differentiated pulmonary fibroblast cells, suggesting that endogenous RGS2 in pulmonary fibroblast cells should function as an anti-fibrotic gene. Indeed, overexpression of recombinant RGS2 or PFD treatment significantly reduced thrombin-induced collagen production and expression of CTGF in cultured human lung fibroblast cells. Interestingly, PFD treatment selectively upregulates RGS2 expression without effects on the other 20 RGS gene family members in human pulmonary fibroblast cells. More importantly, PFD treatment also increased RGS2 expression in primary pulmonary fibroblast cells isolated from patients with IPF. Thus, it is likely that induction of RGS2 by PFD in pulmonary fibroblast cells contributes, at least in part, to the anti-fibrotic effects of PFD in vivo.

Because PFD has a short half-life of only 2.5 h following oral administration [17], the therapeutically effective doses of PFD for human IPF and bleomycin-induced mouse pulmonary fibrosis are very high (>30 mg/kg, daily) [17, 18]. Such high doses can cause significant side effects that in turn limit therapeutic effectiveness [16]. Theoretically, lowering the dose of PFD could result in reduction of side effects. In the current study, we administered PFD by nose-only nebulization at a dose of 0.35 mg/kg. Our data show that PFD can be an effective anti-fibrotic agent at much lower concentrations if the drug is administered by inhalation directly to airways, at least in mice. Thus developing an effective inhaled delivery form of PFD could improve its safety and efficacy in IPF patients.

## Conclusions

In conclusion, our data show that the beneficial effects of PFD in IPF can be explained at least in part by its ability to cause rapid RGS2 upregulation. Moreover, this RGS2 upregulation is both necessary and sufficient for the beneficial effects of PFD to be manifest, both in human cells and in mice. Our data suggest that the maintenance of intracellular RGS2 levels is of paramount importance for preventing fibroblast proliferation and transition to the myofibroblast phenotype that drives IPF, adding to our understanding of IPF pathogenesis and treatment. More importantly, our findings may help in the design of more effective therapeutic strategies to halt and perhaps partially reverse the progression of IPF.

## Abbreviations

[Ca^2+^]_i,_ intracellular Ca^2+^; BrdU_,_ 5-bromo-2’-deoxyuridine; C_dyn,_ dynamic compliance; CPHLF_,_ control primary human lung fibroblast; C_T,_ Threshold cycle; CTGF_,_ connective tissue growth factor; DAPI_,_ 4’,6-diamidino-2-phenylindole; DMEM_,_ Dulbecco's modified eagle's medium; DPHLF, diseased primary human lung fibroblast; F12_,_ Ham's F-12 Nutrient Mixture; FBS_,_ fetal bovine serum; FDA_,_ food and drug administration; GPCRs_,_ G protein-coupled receptors; H&E_,_ hematoxylin and eosin; HFL1_,_ human fetal lung fibroblast; IPF_,_ idiopathic pulmonary fibrosis; PAR1_,_ proteinase-activated receptor 1; PFD_,_ pirfenidone; RGS2_,_ regulator of G-protein signaling 2; α-SMA_,_ α-smooth muscle actin

## Additional file

Additional file 1: Table S1. Differentially expressed genes in PFD treatment lung fibroblast vs. control fibroblast. (DOCX 27 kb)
