# Supplementary material for: Upregulation of RGS2: a new mechanism for pirfenidone amelioration of pulmonary fibrosis
Source: Respir Res. 2016 Aug 22;17(1):103. doi: 10.1186/s12931-016-0418-4 (PMC4994235; doi:10.1186/s12931-016-0418-4)
Supplement: Additional file 1: Table S1. — Differentially expressed genes in PFD treatment lung fibroblast vs. control fibroblast. (DOCX 27 kb) [file 12931_2016_418_MOESM1_ESM.docx]

**Additional file 1 Table S1 Differentially expressed genes in PFD treatment lung fibroblast vs. control fibroblast**

| **Transcript Cluster ID** | **Gene Symbol** | **Description** | **Fold Change** |
| --- | --- | --- | --- |
|  | **Upregulated in PFD treatment vs. Control** |  |  |
| 17002846 | DUSP1 | dual specificity phosphatase 1 | 9.16 |
| 16864983 | ZNF331 | zinc finger protein 331 | 8.12 |
| 17087517 | NR4A3 | nuclear receptor subfamily 4, group A, member 3 | 7.35 |
| 16903897 | NR4A2 | nuclear receptor subfamily 4, group A, member 2 | 7.17 |
| 16705961 | DDIT4 | DNA-damage-inducible transcript 4 | 6.26 |
| 16675323 | RGS2 | regulator of G-protein signaling 2, 24KD | 6.15 |
| 16917004 | GPCPD1 | glycerophosphocholine phosphodiesterase GDE1 homolog (S. cerevisiae) | 5.03 |
| 16856803 | GADD45B | growth arrest and DNA-damage-inducible, beta | 4.98 |
| 16926200 | SIK1 | salt-inducible kinase 1 | 4.86 |
| 17005072 | RNU6-522P | RNA, U6 small nuclear 522, pseudogene | 4.84 |
| 16870367 | KIAA1683 | KIAA1683 | 4.5 |
| 17095703 | NFIL3 | nuclear factor, interleukin 3 regulated | 4.16 |
| 16751438 | NR4A1 | nuclear receptor subfamily 4, group A, member 1 | 3.96 |
| 16912362 | ID1 | inhibitor of DNA binding 1, dominant negative helix-loop-helix protein | 3.88 |
| 17079210 | GEM | GTP binding protein overexpressed in skeletal muscle | 3.87 |
| 17022736 | TUBE1 | tubulin, epsilon 1 | 3.45 |
| 16858592 | ZNF844 | zinc finger protein 844 | 3.43 |
| 16666055 | CTH | cystathionine gamma-lyase; cystathionase (cystathionine gamma-lyase) | 3.31 |
| 16880414 | REL | v-rel avian reticuloendotheliosis viral oncogene homolog; v-rel reticuloendotheliosis viral oncogene homolog (avian) | 3.26 |
| 16688210 | MIR3671 | microRNA 3671 | 3.12 |
| 16849400 | SOCS3 | suppressor of cytokine signaling 3 | 3.1 |
| 16677278 | ATF3 | activating transcription factor 3 | 3.1 |
| 16840846 | PER1; MIR6883 | period circadian clock 1; microRNA 6883; period homolog 1 (Drosophila) | 2.96 |
| 16692764 | LOC100289061; RP11-54A4.2 | uncharacterized LOC100289061; novel transcript, antisense to ADAMTSL4 | 2.93 |
| 16970536 | HSPA4L | heat shock 70kDa protein 4-like | 2.87 |
| 17067182 | RP11-14I17.2 | novel transcript | 2.83 |
| 16661544 | SESN2 | sestrin 2 | 2.77 |
| 16858509 | ZNF627 | zinc finger protein 627 | 2.76 |
| 17076170 | RNF122 | ring finger protein 122 | 2.73 |
| 17025417 | AGPAT4 | 1-acylglycerol-3-phosphate O-acyltransferase 4; 1-acylglycerol-3-phosphate O-acyltransferase 4 (lysophosphatidic acid acyltransferase, delta) | 2.72 |
| 16998059 | ARRDC3 | arrestin domain containing 3 | 2.7 |
| 16863287 | FOSB | FBJ murine osteosarcoma viral oncogene homolog B | 2.66 |
| 17120240 | LOC100287934; LOC101930657; RP11-206L10.9 | uncharacterized LOC100287934; uncharacterized LOC101930657; novel transcript | 2.64 |
| 16980451 | PRMT9; PRMT10 | protein arginine methyltransferase 9; protein arginine methyltransferase 10 (putative) | 2.61 |
| 16891176 | ANKZF1 | ankyrin repeat and zinc finger domain containing 1 | 2.61 |
| 16804409 | RP11-815J21.1 | novel transcript, sense_intronic to AKAP13 | 2.58 |
| 16858607 | ZNF788 | zinc finger family member 788 | 2.53 |
| 17118792 | MIR612; NEAT1 | microRNA 612; nuclear paraspeckle assembly transcript 1 (non-protein coding) | 2.5 |
| 17025438 | AGPAT4-IT1 | AGPAT4 intronic transcript 1 (non-protein coding) | 2.5 |
| 16770664 | TBX5 | T-box 5 | 2.44 |
| 16703642 | MAP3K8 | mitogen-activated protein kinase kinase kinase 8 | 2.41 |
| 16884335 | BCL2L11 | BCL2-like 11 (apoptosis facilitator) | 2.41 |
| 16759676 | ZNF10 | zinc finger protein 10 | 2.37 |
| 16696434 | LOC100506023 | uncharacterized LOC100506023 | 2.35 |
| 17022362 | SESN1 | sestrin 1 | 2.34 |
| 16748477 | CDKN1B | cyclin-dependent kinase inhibitor 1B (p27, Kip1) | 2.33 |
| 17120236 | LOC100287934; LOC101930657; RP11-206L10.9 | uncharacterized LOC100287934; uncharacterized LOC101930657; novel transcript | 2.28 |
| 16714536 | IPMK | inositol polyphosphate multikinase | 2.23 |
| 17019134 | USP49 | ubiquitin specific peptidase 49 | 2.2 |
| 17124778 | FAM185A; FAM185BP | family with sequence similarity 185, member A; family with sequence similarity 185, member B pseudogene; novel pseudogene | 2.19 |
| 16716239 | GLUD1 | glutamate dehydrogenase 1 | 2.18 |
| 17120092 | LINC01359; RP11-182I10.3 | long intergenic non-protein coding RNA 1359; novel transcript | 2.17 |
| 17102387 | GK-IT1 | GK intronic transcript 1 (non-protein coding); novel transcript | 2.17 |
| 16851022 | CHMP1B | charged multivesicular body protein 1B; chromatin modifying protein 1B | 2.13 |
| 16998515 | LOC100133050 | glucuronidase, beta pseudogene | 2.13 |
| 16805230 | CHD2 | chromodomain helicase DNA binding protein 2 | 2.12 |
| 16959862 | RP11-438D8.2 | novel transcript, antisense to ZBTB38 & ACPL2 | 2.12 |
| 17053892 | INSIG1 | insulin induced gene 1 | 2.12 |
| 16703242 | OTUD1 | OTU deubiquitinase 1; OTU domain containing 1 | 2.12 |
| 16679923 | LOC101928706; LOC101929823; RP5-857K21.4; RP4-669L17.10 | uncharacterized LOC101928706; uncharacterized LOC101929823; novel transcript | 2.09 |
| 17010991 | CASP8AP2 | caspase 8 associated protein 2 | 2.08 |
| 16755263 | RNU6-808P | RNA, U6 small nuclear 808, pseudogene | 2.07 |
| 16709108 | MXI1 | MAX interactor 1, dimerization protein; MAX interactor 1 | 2.06 |
| 17050455 | FOXP2 | forkhead box P2 | 2.06 |
| 17025937 | PHF10 | PHD finger protein 10 | 2.06 |
| 17056137 | HOXA5 | homeobox A5 | 2.06 |
| 16744770 | SIK3-IT1 | SIK3 intronic transcript 1 (non-protein coding); novel transcript | 2.06 |
| 16753543 | MIR548C; MIR548Z | microRNA 548c; microRNA 548z | 2.06 |
| 17123920 | LOC100289230 | uncharacterized LOC100289230 | 2.05 |
| 16688792 | RNA5SP22 | RNA, 5S ribosomal pseudogene 22 | 2.04 |
| 16837348 | MAP2K6 | mitogen-activated protein kinase kinase 6 | 2.03 |
| 16849574 | LOC100653515; CTD-2373H9.6 | differential display clone 8; novel protein | 2.02 |
| 16673268 | MGST3 | microsomal glutathione S-transferase 3 | 2.02 |
| 16713307 | RNU6-1167P | RNA, U6 small nuclear 1167, pseudogene | 2.01 |
| 17124774 | FAM185A; FAM185BP | family with sequence similarity 185, member A; family with sequence similarity 185, member B pseudogene; novel pseudogene | 2.01 |
| 17062502 | POT1 | protection of telomeres 1; POT1 protection of telomeres 1 homolog (S. pombe) | 2 |
|  | **Downregulated in PFD treatment vs. Control** |  |  |
| 17110365 | MIR221 | microRNA 221 | 7.93 |
| 17110367 | MIR222 | microRNA 222 | 7.08 |
| 17097643 | TNFSF15 | tumor necrosis factor (ligand) superfamily, member 15 | 5.33 |
| 16784135 | RNA5SP385 | RNA, 5S ribosomal pseudogene 385 | 5.1 |
| 16675840 | RNU6-501P | RNA, U6 small nuclear 501, pseudogene | 5.08 |
| 16901827 | AC017002.2 | novel transcript | 5.05 |
| 16753641 | HMGA2 | high mobility group AT-hook 2 | 4.63 |
| 16784137 | RNU6-301P | RNA, U6 small nuclear 301, pseudogene | 4.13 |
| 17110369 | RP6-99M1.2 | novel transcript | 3.78 |
| 17122666 | LINC00152; MIR4435-1HG; LOC101930489 | long intergenic non-protein coding RNA 152; MIR4435-1 host gene (non-protein coding); uncharacterized LOC101930489; novel transcript | 3.67 |
| 17122664 | LINC00152; LOC101930489; MIR4435-1HG | long intergenic non-protein coding RNA 152; uncharacterized LOC101930489; MIR4435-1 host gene (non-protein coding); novel transcript | 3.55 |
| 16688368 | MIR1262 | microRNA 1262 | 3.31 |
| 17122654 | LINC00152; MIR4435-1HG; LOC101930489 | long intergenic non-protein coding RNA 152; MIR4435-1 host gene (non-protein coding); uncharacterized LOC101930489; novel transcript | 3.15 |
| 16884523 | SLC20A1 | solute carrier family 20 (phosphate transporter), member 1 | 3.09 |
| 16723653 | CD44; RP1-68D18.2 | CD44 molecule (Indian blood group); novel transcript | 2.82 |
| 16724663 | MIR3161 | microRNA 3161 | 2.76 |
| 16684898 | RN7SKP16 | RNA, 7SK small nuclear pseudogene 16 | 2.76 |
| 16697654 | MIR181A1HG | MIR181A1 host gene (non-protein coding); novel transcript | 2.74 |
| 16713309 | FZD8; MIR4683 | frizzled class receptor 8; frizzled homolog 8 (Drosophila); microRNA 4683 | 2.73 |
| 17062945 | LINC-PINT; MIR29A; MIR29B1; AC058791.1 | long intergenic non-protein coding RNA, p53 induced transcript; microRNA 29a; microRNA 29b-1; novel transcript | 2.68 |
| 16768297 | DUSP6 | dual specificity phosphatase 6 | 2.58 |
| 16754410 | RN7SKP172 | RNA, 7SK small nuclear pseudogene 172 | 2.57 |
| 17110372 | FLJ25917; RP5-1158E12.3 | uncharacterized LOC401585; novel transcript | 2.53 |
| 17004903 | EDN1 | endothelin 1 | 2.49 |
| 16767118 | LOC100129940; RP11-366L20.2 | uncharacterized LOC100129940; novel protein | 2.46 |
| 16943241 | COL8A1 | collagen, type VIII, alpha 1 | 2.43 |
| 16740630 | FOSL1 | FOS-like antigen 1 | 2.43 |
| 17125954 | MIR3605 | microRNA 3605 | 2.42 |
| 16716779 | LOC101927063; RP11-310E22.4 | uncharacterized LOC101927063; novel transcript, antisense to C10orf129 | 2.41 |
| 16732394 | OAF | OAF homolog (Drosophila) | 2.38 |
| 17076618 | CTD-3080F16.3 | novel transcript | 2.37 |
| 17124806 | LINC-PINT; MIR29A; MIR29B1; AC058791.1 | long intergenic non-protein coding RNA, p53 induced transcript; microRNA 29a; microRNA 29b-1; novel transcript | 2.34 |
| 17100639 | MT-TF | mitochondrially encoded tRNA phenylalanine | 2.33 |
| 17081215 | ASAP1-IT1 | ASAP1 intronic transcript 1 (non-protein coding) | 2.25 |
| 17122656 | LINC00152; MIR4435-1HG; LOC101930489 | long intergenic non-protein coding RNA 152; MIR4435-1 host gene (non-protein coding); uncharacterized LOC101930489; novel transcript | 2.25 |
| 16799724 | DLL4 | delta-like 4 (Drosophila) | 2.24 |
| 17102755 | DDX3X | DEAD (Asp-Glu-Ala-Asp) box helicase 3, X-linked; DEAD (Asp-Glu-Ala-Asp) box polypeptide 3, X-linked | 2.23 |
| 16754402 | RP11-54A9.1 | novel transcript | 2.22 |
| 16715699 | C10orf55 | chromosome 10 open reading frame 55 | 2.21 |
| 16742742 | MIR708 | microRNA 708 | 2.15 |
| 17001063 | SPRY4 | sprouty homolog 4 (Drosophila) | 2.14 |
| 17125740 | RBMY2QP | RNA binding motif protein, Y-linked, family 2, member Q pseudogene; RNA binding motif protein, Y-linked, family 1, member A1 (RBMY1A1) pseudogene | 2.13 |
| 17100671 | MT-TR | mitochondrially encoded tRNA arginine | 2.1 |
| 17117397 | LOC400743; RP11-380J14.1 | uncharacterized LOC400743; novel transcript | 2.06 |
| 16919769 | NCOA5 | nuclear receptor coactivator 5 | 2.06 |
| 16971139 | ABCE1 | ATP-binding cassette, sub-family E (OABP), member 1 | 2.05 |
| 17000724 | HBEGF | heparin-binding EGF-like growth factor | 2.05 |
| 16970859 | RNU6-531P | RNA, U6 small nuclear 531, pseudogene | 2.05 |
| 17122662 | LINC00152; MIR4435-1HG; LOC101930489 | long intergenic non-protein coding RNA 152; MIR4435-1 host gene (non-protein coding); uncharacterized LOC101930489; novel transcript | 2.04 |
